# Supplementary material for: Hybrid photoacoustic and fast super-resolution ultrasound imaging
Source: Nat Commun. 2023 Apr 18;14:2191. doi: 10.1038/s41467-023-37680-w (PMC10113238; doi:10.1038/s41467-023-37680-w)
Supplement: Supplementary file 1 — Supplementary Information [file 41467_2023_37680_MOESM1_ESM.pdf]

Supplementary Information for

Hybrid Photoacoustic and Fast Super-Resolution Ultrasound Imaging

Shensheng Zhao, Jonathan Hartanto, Ritin Joseph, Cheng-Hsun Wu, Yang Zhao, Yun-Sheng Chen*

*Corresponding author: yunsheng@illinois.edu

Supplementary Information Contents

Supplementary Notes.

Supplementary Note 1: Localization limitation of SC and PSF-CC algorithms

Supplementary Note 2: Localization performance of SC and PSF-CC algorithms with different microbubble densities

Supplementary Figures.

Supplementary Figure S1. Two microbubbles localization using SC and PSF-CC.

Supplementary Figure S2. Multiple microbubbles localization using SC and PSF-CC.

Supplementary Figure S3. Separation distance distribution of a microbubble to its nearest neighbor with a concentration of 16 (red) and 104 (blue) microbubbles/mm^2.

Supplementary Figure S4. Localization error of the SC method as a function of microbubble densities.

Supplementary Figure S5. Comparison of various SC methods with the traditional PSF-CC method.

Supplementary Figure S6. Characterization of microbubbles (MB).

Supplementary Figure S7. Comparison of SC-ULM and PSF-CC ULM in a mouse kidney.

Supplementary Figure S8. Motion correction in super-resolution kidney imaging.

Supplementary Figure S9. Fourier ring correlation (FRC) resolution measurement curves of the SC-ULM and PSF-CC ULM images at various data acquisition (DAQ) times.

Supplementary Figure S10. The resolution of super-resolution ultrasound kidney image as a function of the data acquisition (DAQ) time.

Supplementary Figure S11. SC-ULM image of the kidney before and after in-plane motion correction.

Supplementary Figure S12. SC-ULM shows the dynamics.

Supplementary Figure S13. Representative PA/ULM images reveal renal functional information of a mouse during an oxygen challenge test.

Supplementary Tables.

Supplementary Table 1. The data acquisition and processing time of dual-modal PA/ULM imaging

Supplementary Table 2. The data processing time of different localization algorithms

Supplementary Note 1: Localization limitation of SC and PSF-CC algorithms

To explore the detection limitation of SC and PSF-CC algorithms, we first consider only two microbubbles here. Figure S1 shows an example of two microbubbles separated by different distances in the simulation. When the distance of microbubbles is 25 μm (Figure S1a), both SC and PSF-CC only resolve one position, which means the distance of the microbubbles is beyond the resolution limit of both algorithms. When increasing the distance to 38 μm (Figure S1b), although microbubbles are still overlapped in the ultrasound image, SC can identify both bubbles, while PSF-CC cannot. PSF-CC can only retrieve two locations when the distance is larger than 162 μm (Figure S1c), in which the microbubbles are almost independent and slightly overlapped. The PSF-CC method is based on calculating the cross-correlation coefficient between the PSF pattern and the image. The method identifies the positions of microbubbles by finding the local maximum values of the cross-correlation map. It only compares the PSF pattern with the microbubble pattern pixel by pixel and finds the position with the "highest similarity" (the peak value in the correlation map). In the highly overlapped case (Figures S1a and b), the shape of ultrasound images is similar to a single PSF pattern. Thus only one peak value is found in the correlation map.

The situation will get more complicated when more than two microbubbles are overlapped. For example, Figure S2 shows the identification of three overlapped microbubbles using SC and PSF-CC methods. In Figure S2a, microbubbles are separated by more than 100 μm. Although the correlation map shows several peaks, due to the low correlation value (less than 0.6, the threshold), no position is identified by the PSF-CC method. However, SC can still identify them. When microbubbles get closer (Figure S2b and c), the pattern of the ultrasound image is close to the PSF pattern. Therefore, PSF-CC gets one peak value in the correlation map and retrieves one position.

For SC, in Figure S2c, the distances of every two microbubbles are above the resolution limit (36 μm), but three microbubbles are much closer compared to the distances in Figure S2a and b; only two positions can be retrieved.

Supplementary Note 2: Localization performance of SC and PSF-CC algorithms with different microbubble densities

In the simulation, we assume the microbubble distribution is a random distribution (Gaussian distribution). Statistically, the ratio of the aggregated microbubbles with low bubble densities is lower than the ratio in high-density bubbles. To illustrate the effect, Figure S3 plots the distribution of microbubble separations in two different microbubble densities (16 and 104 microbubbles/mm^2). As expected, the average separation distance of the low-density case is larger than that of the high-density case. The minimum distances between two microbubbles that PSF-CC and SC methods can distinguish are marked as dash lines. Figure S3 shows that in the low-density case (the red curve), the PSF-CC method can identify around one-third of the microbubbles (estimated by integrating the curve in the right side of the PSF-CC dash line), and SC can identify more than 90% of the microbubbles (estimated by integrating the curve in the right side of SC dash line). In the high-density case, the PSF-CC method can barely identify any microbubbles, but SC can still identify around 80% of the microbubbles. This situation only includes two MB overlapped cases. As mentioned in Note 1, the resolution limit of PSF-CC and SC is more complicated to calculate for multiple overlapped microbubbles. In practice, the numbers of aggregated microbubbles, the amplitude of microbubble signals, and the distance between microbubbles are all critical factors that cause the retrieving error.

Supplementary Figures

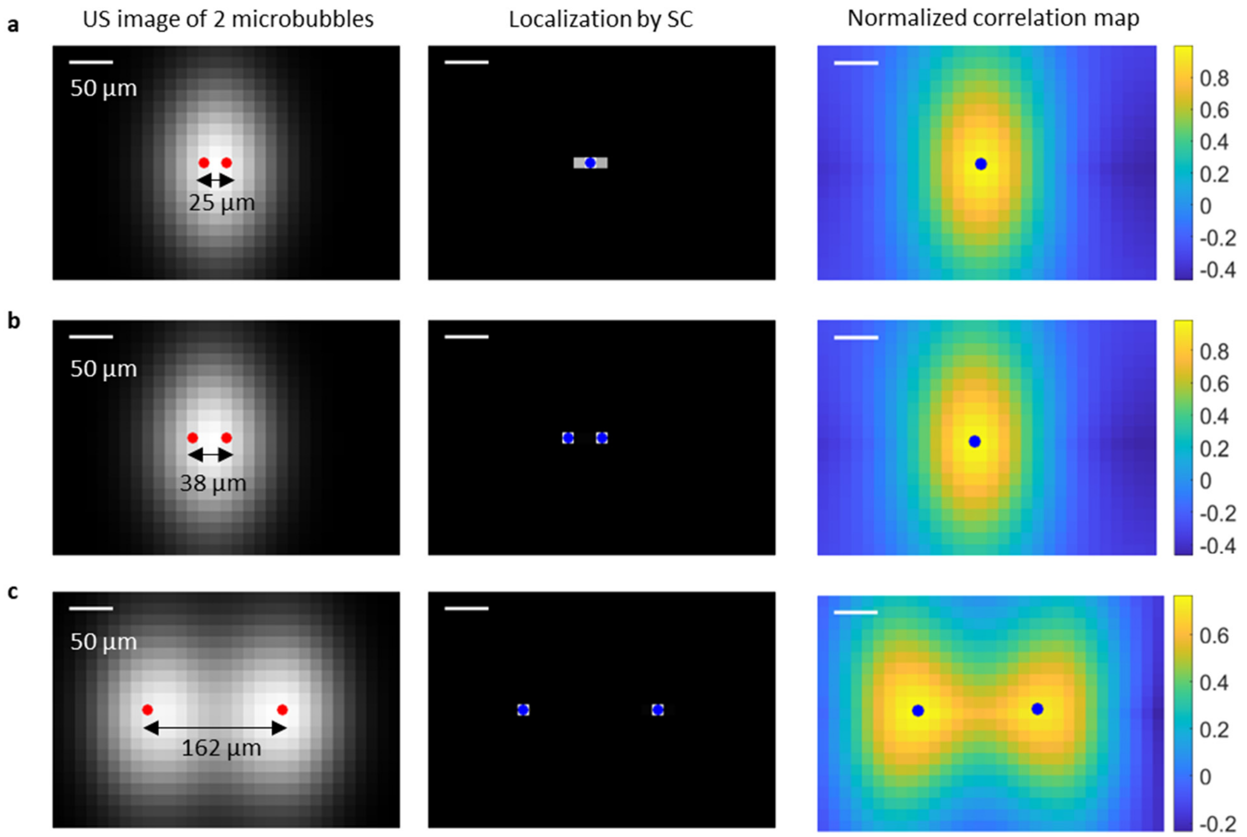

Figure S1| Two microbubbles localization using SC and PSF-CC. Left column: a US image of two microbubbles with different distances; red dots represent the real position of microbubbles. Middle column: the localization image recovered by SC optimization; blue dots represent the recovered microbubbles using SC. Right column: the normalized correlation map generated by PSF-CC; blue dots represent the recovered microbubbles using PSF-CC. (a) The distance of microbubbles is 25 μm, and SC and PSF-CC can only retrieve one position. (b) The distance of microbubbles is 38 μm, SC can retrieve two positions while PSF-CC only retrieves one position. (c) The distance of microbubbles is 162 μm, both SC and PSF-CC can retrieve two positions.

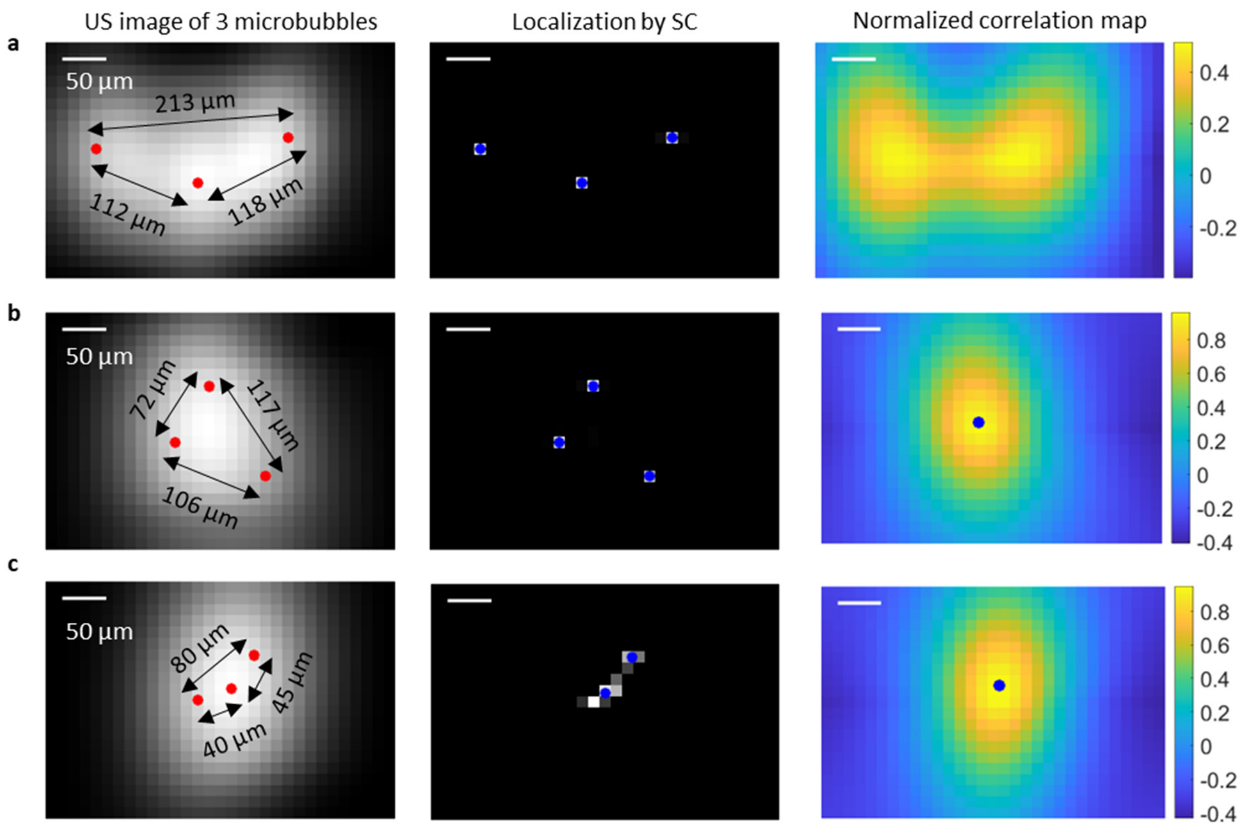

Figure S2| Multiple microbubbles localization using SC and PSF-CC. Left column: a US image of three microbubbles with different distances; red dots represent the real position of microbubbles. Middle column: the localization image recovered by SC optimization; blue dots represent the recovered microbubbles using SC. Right column: the normalized correlation map generated by PSF-CC; blue dots represent the recovered microbubbles using PSF-CC. (a) SC retrieves three positions while PSC-CC retrieves no position because of low correlation indices (threshold is 0.6). (b) SC retrieves three positions while PSC-CC only retrieves one position. (c) SC retrieves two positions while PSC-CC only retrieves one position.

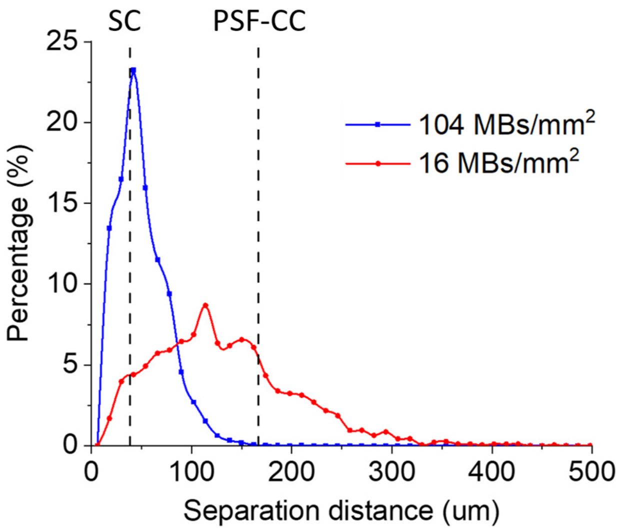

Figure S3| Separation distance distribution of a microbubble to its nearest neighbor with a concentration of 16 (red) and 104 (blue) microbubbles/mm^2. The dashed lines represent the cut-off of the minimum separation in SC and PSF-CC methods.

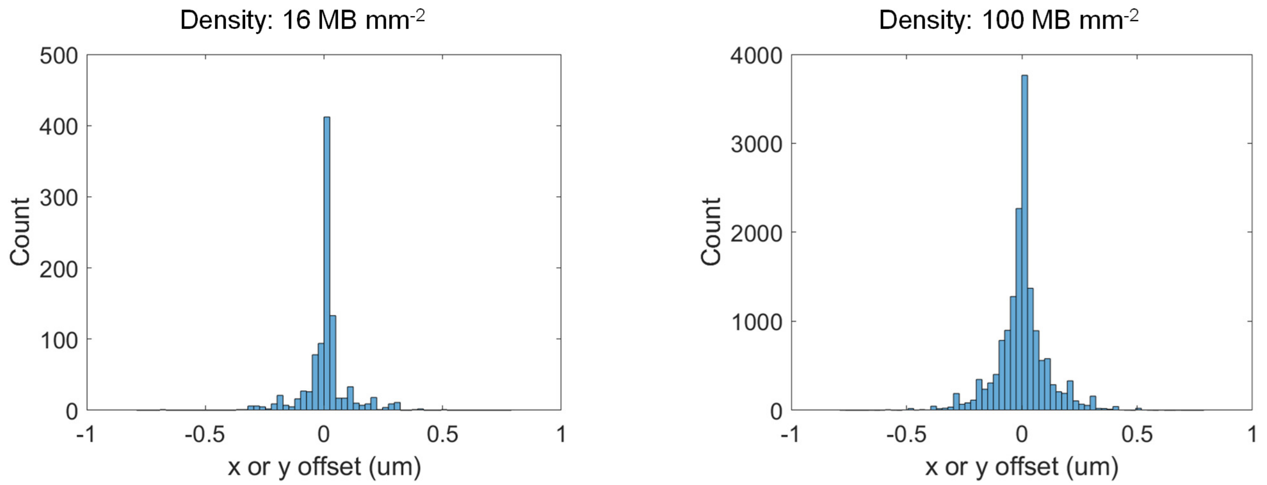

Figure S4| Localization error of the SC method as a function of microbubble densities. The displayed histogram is the summed histogram of the x-offset and y-offset. The x-offset and y-offset are calculated by matching the microbubble positions identified by the SC method and the closest true microbubble positions in the simulation. The two histograms are fitted with Gaussian functions to measure the localization error.

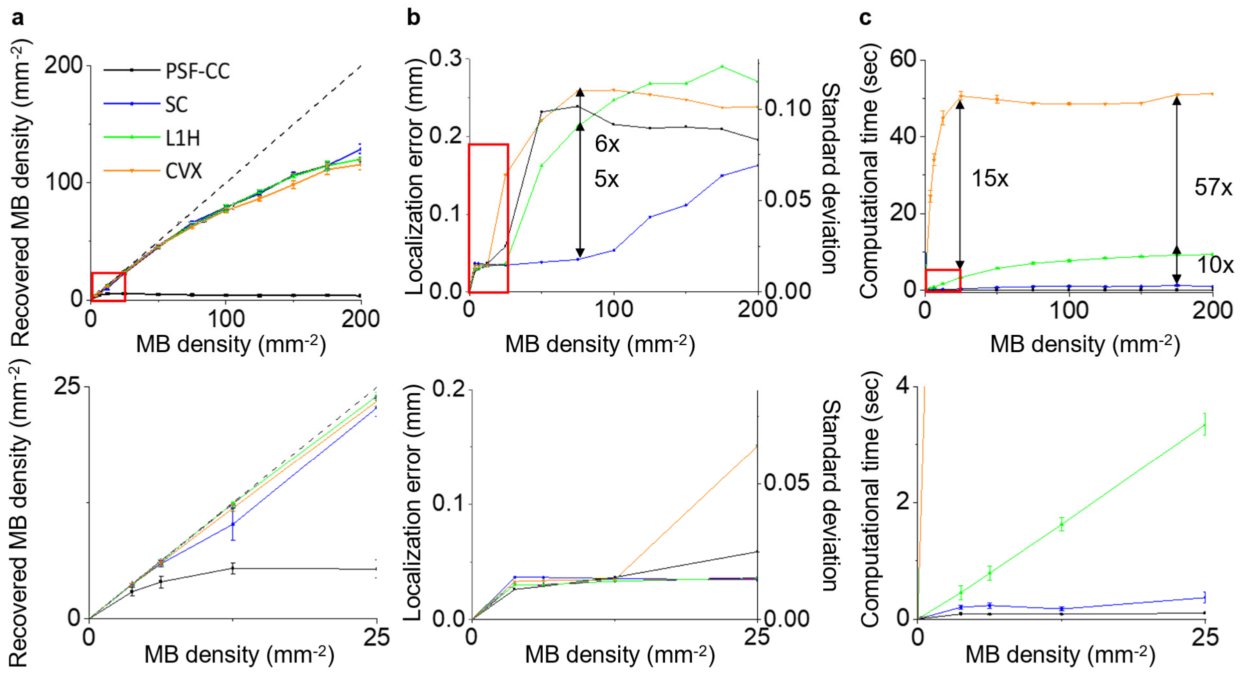

Figure S5| Comparison of various SC methods with the traditional PSF-CC method. The top row is the comparison of recovered microbubble (MB) densities from various methods; the bottom row is the zoom-in area highlighted by the red box. (a) Quantitative analysis of the number of recovered MB versus MB densities. At each MB density, the simulation runs 20 times. The error bars stand for standard deviation (N=20). The dashed line is the reference, representing 100% MB recovery. (b) Quantitative analysis of the localization error versus MB densities. The localization error is measured from the FWHM of the fitted Gaussian function from Figure S1. The standard deviation labeled in the right y-axis is calculated based on Gaussian function properties, which is roughly 1/2.355 of the FWHM. (c) Quantitative analysis of the computational time versus MB densities. The error bars stand for standard deviation (N=20).

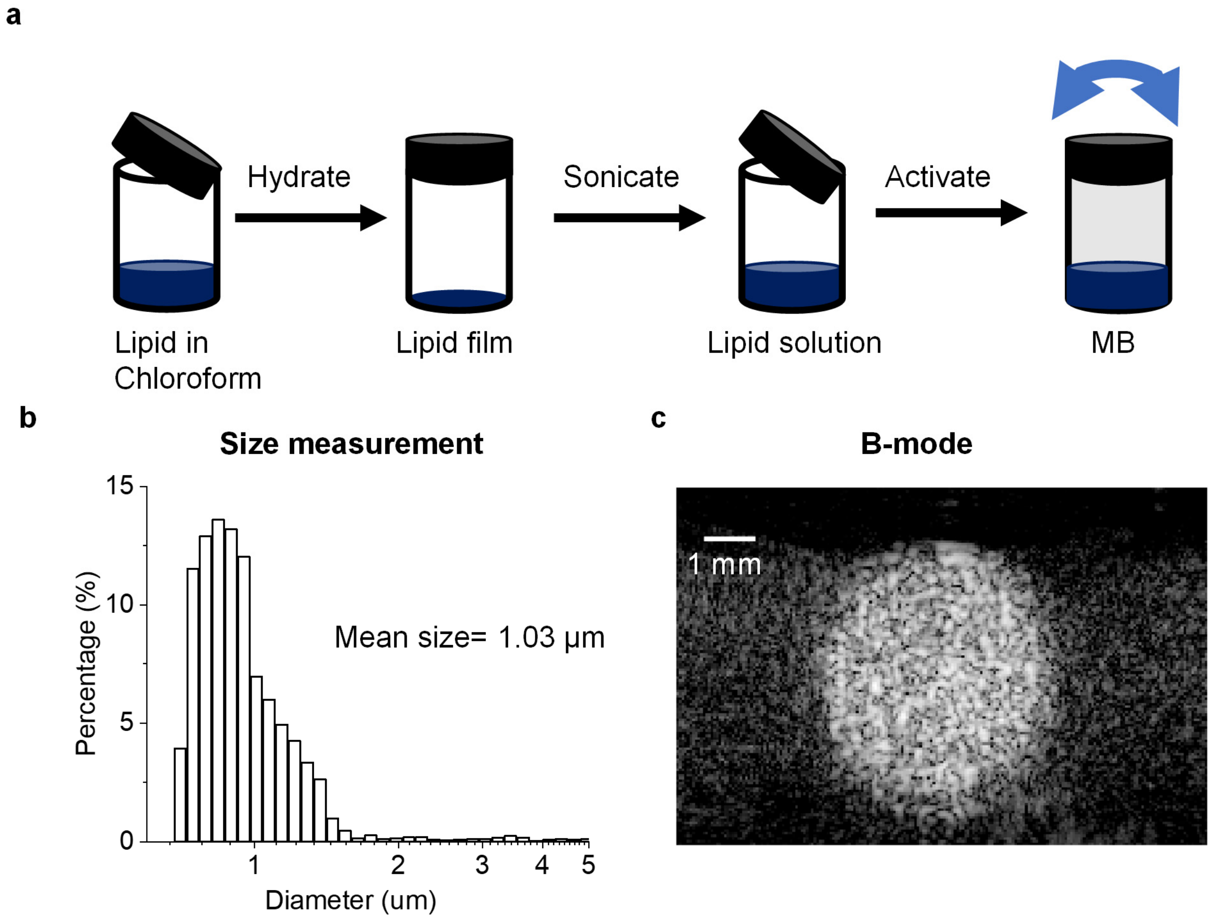

Figure S6| Characterization of microbubbles (MB). (a) The MB synthesis procedure. The lipid/chloroform mixture is hydrated to remove the chloroform. The lipid film is then merged with PBS solution and sonicated. In the activation step, the lipid solution is purged into perfluorobutane and activated by shaking. (b) The size distribution of MB. The mean size is 1.03 µm. (c) Ultrasound image of a MB-embedded inclusion in a tissue-mimicking phantom. The circular area is the inclusion, which shows the ultrasound contrast enhancement compared to the surrounding phantom.

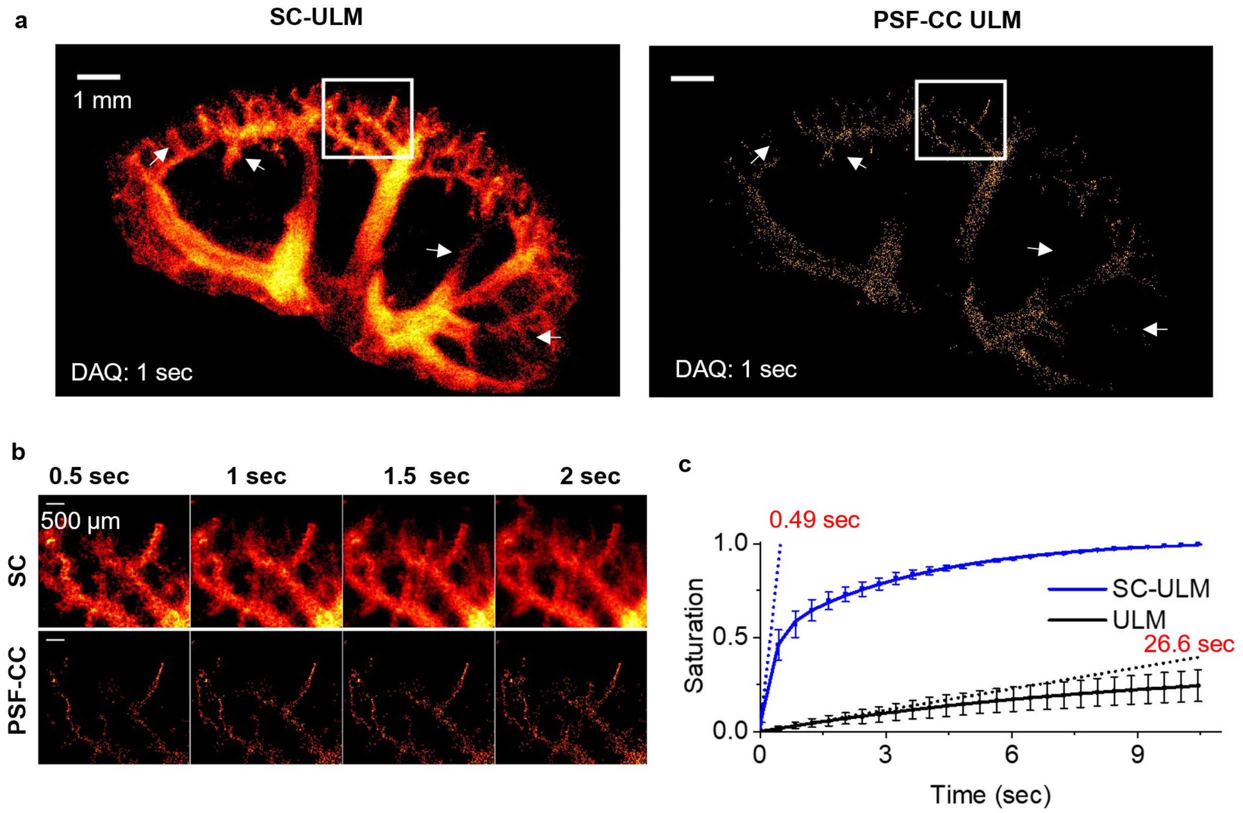

Figure S7| Comparison of SC-ULM and PSF-CC ULM in a mouse kidney. (a) Left to right: SC-ULM and PSF-CC ULM of a mouse kidney in 1 sec of data acquisition (DAQ). (c) Zoom-in view of the vascular structures in SC-ULM (top row) and PSF-CC ULM (bottom row) at various DAQ times ranging from 0.5 seconds to 2 seconds. The area shown here corresponds to the white rectangle in (a). (c) Image saturation as a function of DAQ times in SC-ULM (blue) and PSF-CC ULM images (black), respectively. The average saturation is calculated from 96125 pixels of the three randomly selected regions of interest (ROI) within the vascular structures. The error bars are standard deviations (N = 3). The characteristic time of SC-ULM and PSF-CC ULM is 0.49 sec and 26.6 sec, respectively.

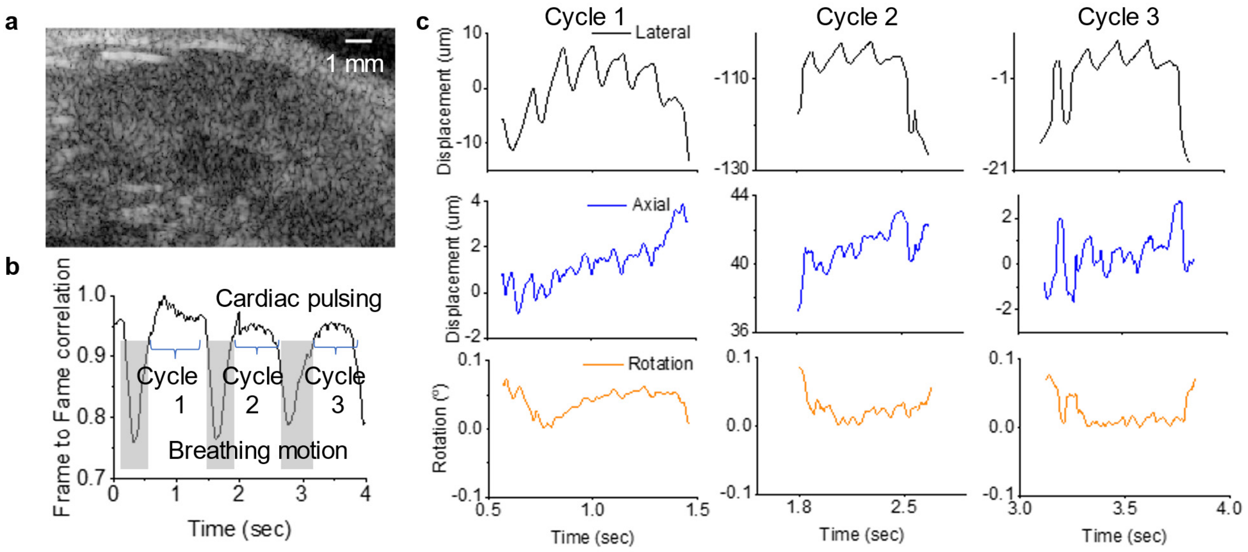

Figure S8| Motion correction in super-resolution kidney imaging. A total of four seconds of data acquisition is shown as a demonstration. (a) The ultrasound image is manually selected as the reference to calculate the frame-to-frame correlation in (b). The 3 complete breathing cycles are identified from the correlation curve. (c) The intra-cycle motions of 3 breathing cycles, including lateral and axial displacements and rotation, are displayed as a function of time, showing the tractable in-plane rigid motions.

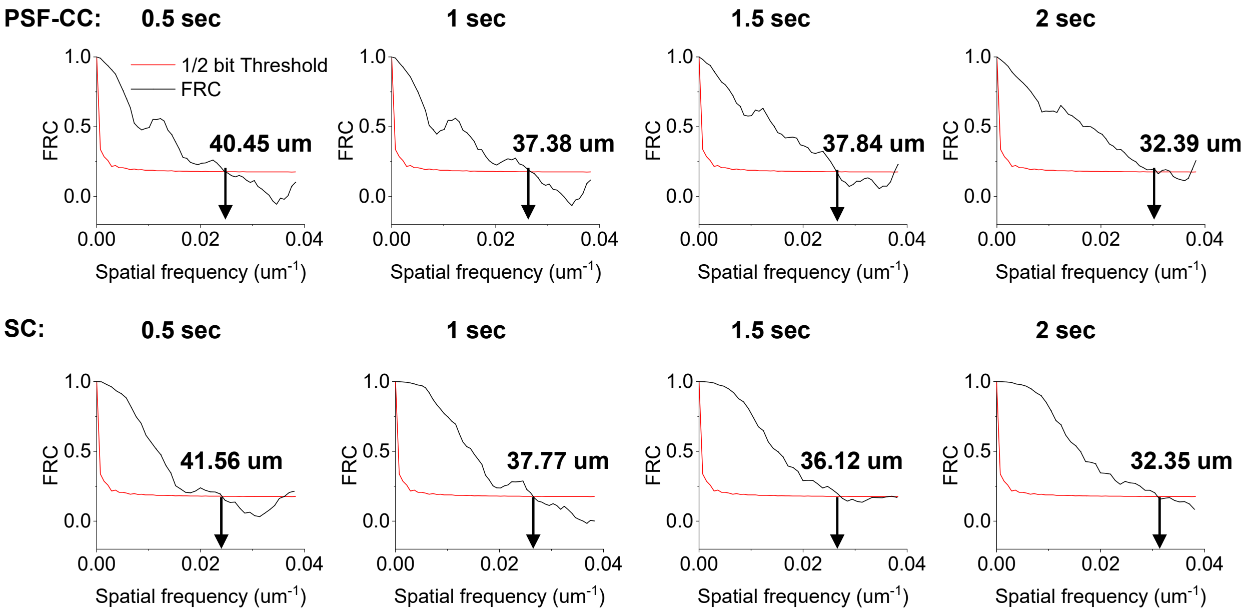

Figure S9| Fourier ring correlation (FRC) resolution measurement curves of the SC-ULM and PSF-CC ULM images at various data acquisition (DAQ) times. The measurement areas are highlighted in the kidney images (Figure S7b).

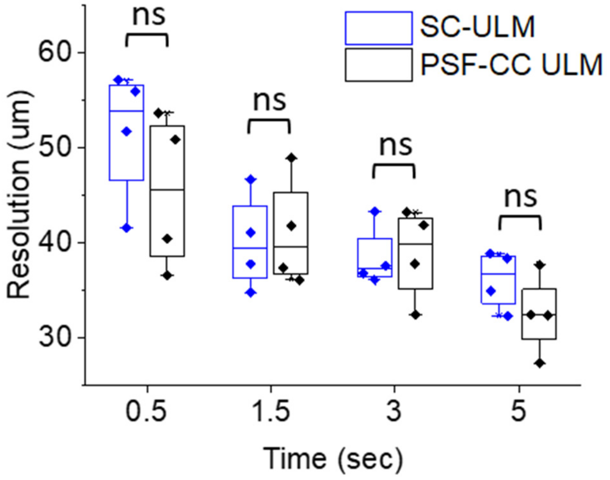

Figure S10| The resolution of super-resolution ultrasound kidney image as a function of the data acquisition (DAQ) time. The resolution is measured using the intersection of the FRC curve and the corresponding half-bit threshold curve. The center line in each box is the median, and the bottom and top edges of the box indicate the 25th and 75th percentiles, respectively. The whiskers range of each box is within 1.5 interquartile. Scatter plots of the data used for the boxplot are overlaid on each boxplot. The t-test shows there is no significant (ns) difference between the resolution of SC-ULM and PSF-CC ULM at the same DAQ time ($p>0.05$).

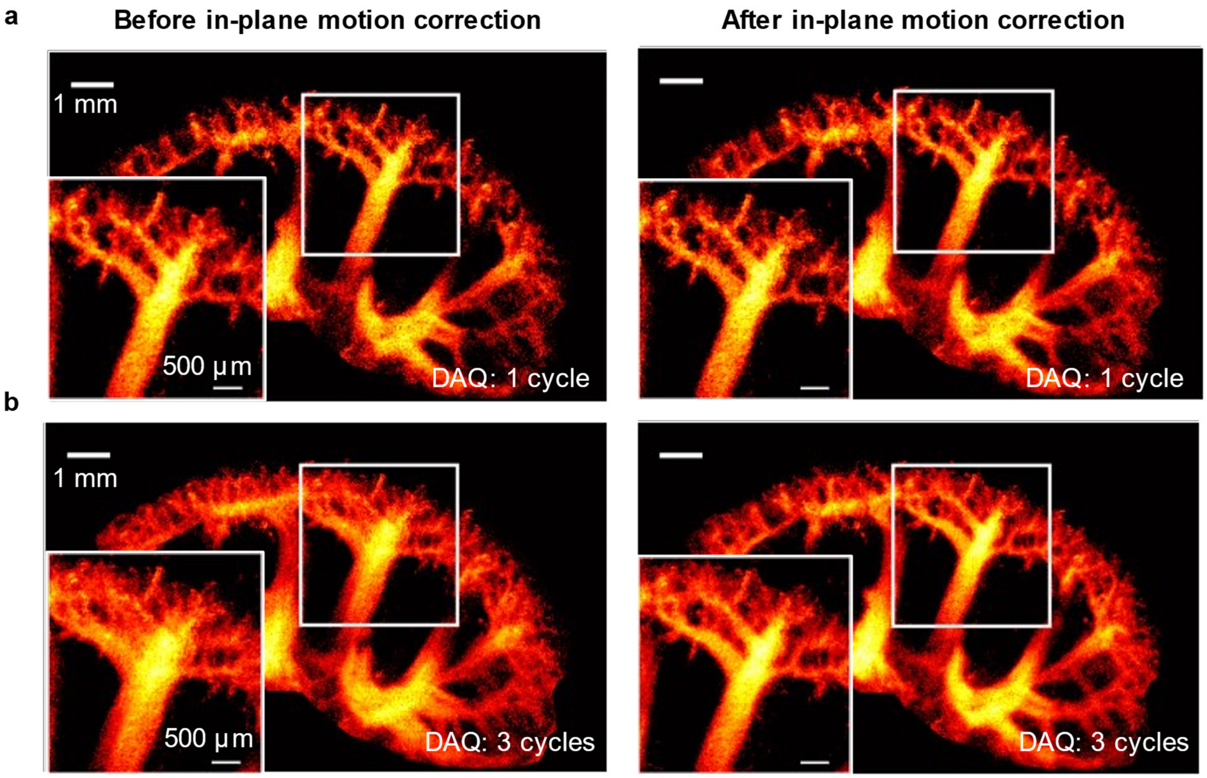

Figure S11| SC-ULM image of the kidney before and after in-plane motion correction. The comparison of SC-ULM images in (a) one breathing cycle and (b) three breathing cycles before and after in-plane motion correction.

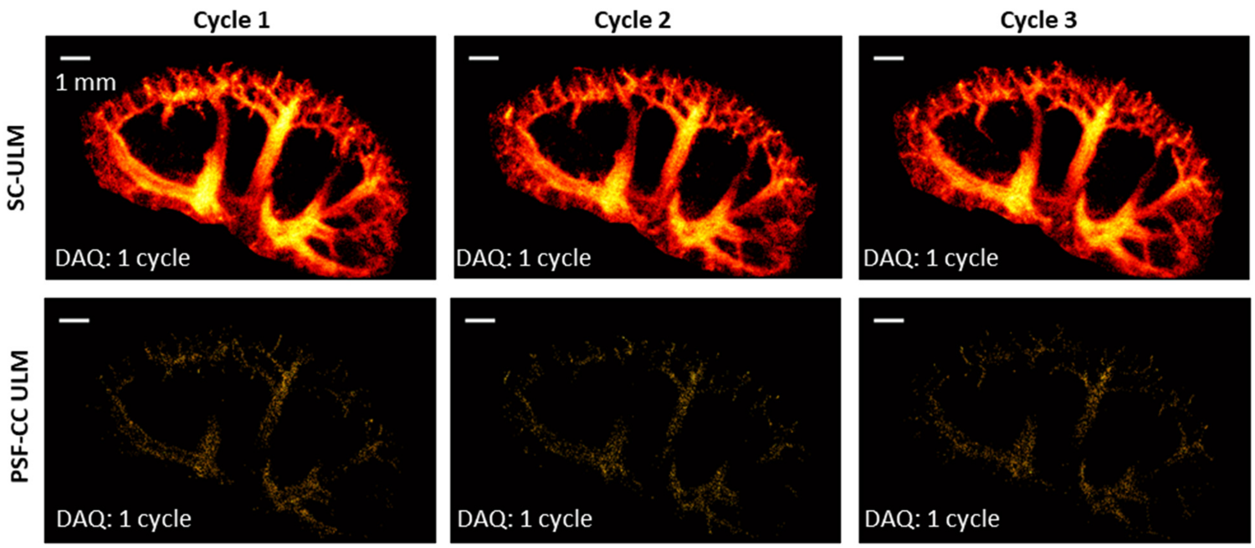

Figure S12| SC-ULM shows the dynamics. The SC-ULM (top) and PSF-CC ULM (bottom) images recorded in three different breathing cycles (the DAQ time for each image is one cycle) after correcting the breathing motion artifacts. SC-ULM enables the tracking of dynamic displacement of kidney vasculature causing respiratory motions. However, PSF-CC ULM is too slow to reconstruct the visible vessel structures within a breathing cycle.

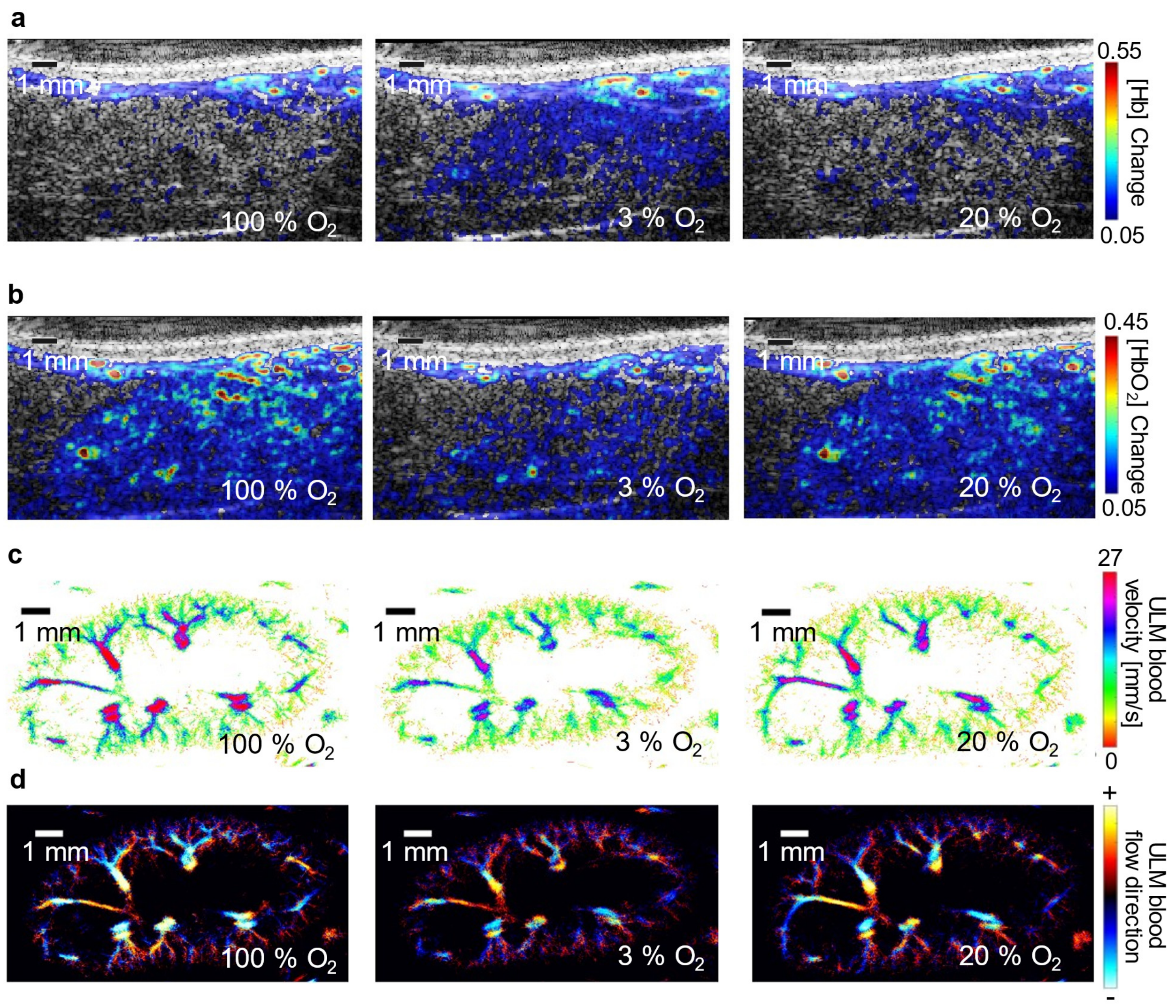

Figure S13| Representative PA/ULM images reveal renal functional information of a mouse during an oxygen challenge test. (a) Changes of renal deoxyhemoglobin, (b) changes of renal oxyhemoglobin, (c) renal blood speed map, (d) directional renal blood flow map at different levels of oxygen inhalations. The oxygen levels were set at 100 %, 3 %, and 20 % during the experiment. The duration of the inhalation at each level is 50 seconds.

SI Table 1. the data acquisition and processing time of dual modal PA/ULM imaging.

	**Data acquisition (sec)**			**PA processing (sec/frame)**		
	PA	US	**Total**	Image reconstruction	Spectrum unmixing	**Total**
**Lymph node** (US image size: 50x135)	0.3	1.5	1.8	0.014	0.537	0.551
**Kidney** (US image size: 60x175)	0.2	0.8	1	0.009	0.494	0.503

SI Table 1. (continued)

	**ULM processing (sec/frame)**				
	Image reconstruction	Clutter filtering	Motion Correction	SC optimization	**Total**
**Lymph node** (US image size: 50x135)	0.014	0.026	0.028	6.2	6.268
**Kidney** (US image size: 60x175)	0.009	0.022	0.026	5.8	5.857

SI Table 2. the data processing time of the localization optimization algorithms.

	**CVX (sec/frame)**	**L1H (sec/frame)**	**FISTA (sec/frame)**
**Lymph node** (US image size: 50x135)	850.1	65.9	6.2
**Kidney** (US image size: 60x175)	545.7	42.3	5.8
